# Cell-Based Reporter Release Assay to Determine the Activity of Calcium-Dependent Neurotoxins and Neuroactive Pharmaceuticals

**DOI:** 10.3390/toxins13040247

**Published:** 2021-03-30

**Authors:** Andrea Pathe-Neuschäfer-Rube, Frank Neuschäfer-Rube, Gerhard P. Püschel

**Affiliations:** Department of Nutritional Biochemistry, Institute of Nutritional Science, University of Potsdam, 14469 Potsdam, Germany; dr.apnr@gmail.com (A.P.-N.-R.); gpuesche@uni-potsdam.de (G.P.P.)

**Keywords:** cell-based assay, neurotoxins, muscarinic acetylcholine receptor, voltage-dependent calcium channels, VGCC

## Abstract

The suitability of a newly developed cell-based functional assay was tested for the detection of the activity of a range of neurotoxins and neuroactive pharmaceuticals which act by stimulation or inhibition of calcium-dependent neurotransmitter release. In this functional assay, a reporter enzyme is released concomitantly with the neurotransmitter from neurosecretory vesicles. The current study showed that the release of a luciferase from a differentiated human neuroblastoma-based reporter cell line (SIMA-hPOMC1-26-GLuc cells) can be stimulated by a carbachol-mediated activation of the Gq-coupled muscarinic-acetylcholine receptor and by the Ca^2+^-channel forming spider toxin α-latrotoxin. Carbachol-stimulated luciferase release was completely inhibited by the muscarinic acetylcholine receptor antagonist atropine and α-latrotoxin-mediated release by the Ca^2+^-chelator EGTA, demonstrating the specificity of luciferase-release stimulation. SIMA-hPOMC1-26-GLuc cells express mainly L- and N-type and to a lesser extent T-type VGCC on the mRNA and protein level. In accordance with the expression profile a depolarization-stimulated luciferase release by a high K^+^-buffer was effectively and dose-dependently inhibited by L-type VGCC inhibitors and to a lesser extent by N-type and T-type inhibitors. P/Q- and R-type inhibitors did not affect the K^+^-stimulated luciferase release. In summary, the newly established cell-based assay may represent a versatile tool to analyze the biological efficiency of a range of neurotoxins and neuroactive pharmaceuticals which mediate their activity by the modulation of calcium-dependent neurotransmitter release.

## 1. Introduction

Disorders in neurotransmitter release are key features of severe neuronal diseases like Parkinson, chronic pain, and depression [1,2,3]. Neurotoxins and neuroactive pharmaceutical substances which affect neurotransmitter release are highly interesting tools in the treatment of neuronal diseases. Bacterial neurotoxins such as botulinum toxins and tetanus toxins block neurotransmitter release by the highly specific proteolytic inactivation of target snare proteins SNAP-25, syntaxin and synaptobrevin (VAMP), which are essential for the fusion of neurosecretory vesicles with the plasma membrane [4]. On the other hand, neurotransmitter release can also be modulated by neurotoxins and neuroactive pharmaceuticals without affecting the activity of these target proteins.

The key step in neurotransmitter release is the surge in the intracellular Ca^2+^-concentration that triggers the fusion of neurosecretory vesicles with the plasma membrane of the presynaptic cell [5,6,7]. The increase of intracellular calcium concentration can be provoked either by an influx of extracellular calcium along the concentration gradient by opening Ca^2+^-channels or by the release from intracellular stores like the sarcoplasmatic reticulum. The entry of extracellular calcium can be stimulated by a depolarization-mediated opening of voltage gated calcium channels (VGCC) or by a neurotoxin mediated de novo formation of Ca^2+^-channels. Activation of Gq-coupled GPCR on the surface of the presynaptic cell will lead to activation of phospholipase C, the formation of second messenger IP3 and therefore release of calcium from intracellular stores.

In addition to proteolytic bacterial neurotoxins, other neurotoxins and neuroactive pharmaceuticals which stimulate or inhibit neurotransmitter release by modulation of intracellular Ca^2+^-concentration are of particular interest in the treatment of neuronal diseases. For this reason, animal derived neurotoxins and synthetic compounds which inhibit the activity of VGCCs or modulate the activation or inhibition of the Gq-coupled muscarinic acetylcholine receptor system were screened for their potential to modulate neurotransmitter release [3,8,9]. The biological activity of these neurotoxins is measured in vivo by mouse lethality assays or in vitro cell culture assays by measuring the modulation of intracellular calcium concentration using fluorogenic calcium dyes. A third approach is the direct quantification of neurotransmitters in cell culture supernatants. Whereas the mouse assay is ethically problematic, determination of the intracellular calcium concentration in cells might not directly reflect the neurotoxic potential of a compound. On the other hand, measurement of neurotransmitters in cell culture supernatants is time consuming and not suitable for high throughput screening.

In this study, a completely different approach to measure the biologic activity of a variety of agonists, antagonists and toxins was used: The neuronal cell line SIMA was stably transfected with a plasmid coding for Gaussia princeps luciferase (GLuc), which was N-terminally extended with the leader sequence of human proopiomelanocortin (hPOMC1-26) that redirects the GLuc into neuro-secretory vesicles. From these vesicles, GLuc was released upon depolarization of the cells into the cell culture supernatant together with neurotransmitters. In proof of principle, it was recently shown that the depolarization-dependent release was efficiently inhibited by BoNT/A and to a lesser extent by BoNT/B and tetanus toxin [10,11]. The goal of this study was to analyze whether this assay is also suitable to detect the activity of other neurotoxins and neuroactive pharmaceuticals which act by stimulation or inhibition of calcium-dependent neurotransmitter release. To this end, the modulation of GLuc release by the muscarinic acetylcholine receptor agonist carbachol and its antagonist atropine, as well as the Ca^2+^-channel forming neurotoxin α-latrotoxin and inhibitors of VGCCs was analyzed in SIMA-hPOMC1-26-GLuc cells.

## 2. Results

### 2.1. Suitability for Testing Compounds Leading to Neurotransmitter Release by an Increase of Intracellular Ca^2+^-Concentration from Intracellular Stores

**Figure 1:**
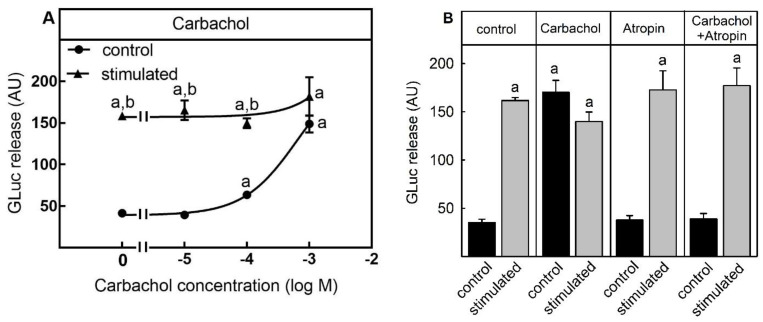
Carbachol-dependent stimulation of luciferase release. SIMA cells stably expressing hPOMC1-26 GLuc were cultured and differentiated as described in the methods section. After removing the medium, cells were washed with fresh medium and incubated in differentiation medium in the absence or presence of 500 µM atropine for 10 min. Cells were then incubated for three minutes with non-depolarizing (Na^+^, control) or depolarizing (K^+^, stimulated) balanced salt solution in the presence of different carbachol concentrations (**A**) or 1 mM carbachol −/+ 500 µM atropine (**B**). Cell culture supernatants were centrifuged and luciferase activity was determined in the cell culture supernatants. Values are means ± SEM of at least three independent experiments. Statistics: Student’s t-test for unpaired samples, a: > control buffer without carbachol: b: > control buffer with the respective carbachol concentration; *p* < 0.05.

To assess the suitability of the luciferase release assay for testing compounds which provoke neurotransmitter release because of an increase of intracellular calcium-concentration from intracellular pools, cells were stimulated with the M3-muscarinic acetylcholine receptor agonist carbachol. Activation of the M3 receptor leads to an activation of Gq protein and therefore an IP3-mediated release of Ca^2+^ from the endoplasmic reticulum. When cells were incubated with a non-depolarizing Na^+^-containing buffer, a certain amount of luciferase activity was released into the cell culture supernatant. (Figure 1A). This unspecific release was significantly increased by stimulation with 100 µM and 1000 µM carbachol. At a concentration of 1000 µM carbachol luciferase release into the medium was nearly identical to the release stimulated by a K^+^-containing depolarizing buffer, which was 3–4-fold higher than the unspecific release. Carbachol did not influence luciferase release induced by K^+^-depolarization buffer. To test if the stimulation of luciferase release was specific for the activation of the muscarinic acetylcholine receptor, cells were treated with the muscarinic acetylcholine receptor antagonist atropine before and during the carbachol stimulation. While 500 µM atropine did not influence the luciferase release by K^+^-mediated depolarization, the carbachol-stimulated release was completely abolished by atropine (Figure 1B).

Thus, the cell-based assay was suitable to determine the neurotransmitter release stimulated by a liberation of calcium from intracellular stores.

### 2.2. Suitability for Testing Compounds Leading to Neurotransmitter Release by an Increase of Intracellular Ca^2+^-Concentration by Ca^2+^-Channel-Forming Neurotoxins

**Figure 2:**
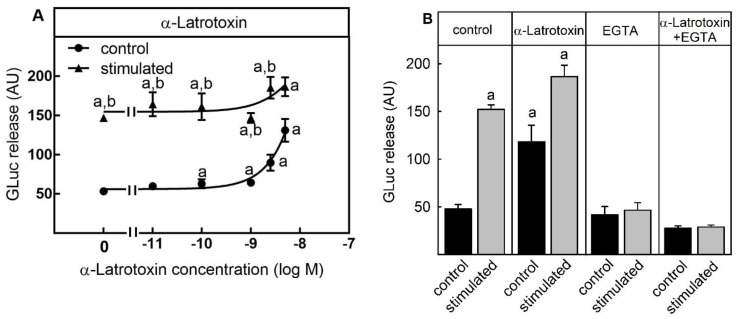
α-Latrotoxin-dependent stimulation of luciferase release. SIMA cells stably expressing hPOMC1-26 GLuc were cultured and differentiated as described in the methods section. After removing the medium, cells were washed with fresh medium and incubated in differentiation medium for 10 min. Cells were then incubated for five minutes with non-depolarizing (Na^+,^ control) or depolarizing (K^+^, stimulated) balanced salt solution in the presence of different α-latrotoxin concentrations (**A**) or 5 nM α-latrotoxin −/+ 10 mM EGTA (**B**). Cell culture supernatants were centrifuged, and luciferase activity was determined in the cell culture supernatants. Values are means ± SEM of at least three independent experiments. Statistics: Student’s t-test for unpaired samples, a: > control buffer without α-latrotoxin: b: > control buffer with the respective α-latrotoxin concentration; *p* < 0.05.

In addition to depolarization or Gq-coupled receptor stimulation, neurotransmitter release can also be activated by the action of Ca^2+^ pore forming LTX-neurotoxins produced by black-widow spiders from the latrodectus family [12]. The mammalian-specific α-latrotoxin (α-LTX) is a relatively big protein (1381 AA) which can bind the presynaptic cell adhesion protein neurexin, leading to the formation of a new Ca^2+^-channel in the membrane of the presynaptic cells. Since this Ca^2+^-channel is permanently open, extracellular Ca^2+^ can enter the cell along the concentration gradient, leading to neurotransmitter release and therefore the permanent depolarization of postsynaptic cells. For this reason, the potential of α-LTX to stimulate luciferase release from SIMA-hPOMC1-26-GLuc cells was tested. α-LTX significantly and dose-dependently increased luciferase release under control conditions up from 0.1 nM α-LTX (Figure 2A). At a concentration of 10 nM, α-LTX stimulated luciferase release into the medium was as high as the release stimulated by a K^+^-containing depolarization buffer (3-fold). Similar to carbachol, α-LTX did not influence luciferase release induced by K^+^-depolarization. To test if the α-LTX-mediated stimulation of luciferase release was Ca^2+^-dependent, cells were treated with the Ca^2+^-chelator EGTA during the stimulation by K^+^-depolarization or by α-LTX. Whereas EGTA did not reduce luciferase release under control conditions, both the K^+^-dependent depolarization and α-LTX-mediated release was completely blocked by EGTA (Figure 2B).

Thus, the cell-based assay was also suitable to determine the neurotransmitter release stimulated by the entry of extracellular Ca^2+^ via Ca^2+^-channel forming α-LTX.

### 2.3. Suitability for Testing Compounds Leading to an Inhibition of Neurotransmitter Release by Blocking Voltage-Gated Ca^2+^-Channels (VGCC)

Many neurotoxins and neuroactive compounds act as inhibitors of voltage-gated-calcium channels (VGCC). Voltage-gated calcium channels are activated by action potential-mediated depolarization. Therefore, calcium influx triggers synaptic vesicle exocytosis leading to release of excitatory neurotransmitters [13]. VGCCs can be classified based on their voltage activation characteristics as high or low-voltage activated channels [14]. The VGCCs can be further subdivided based on their structural similarities of the channel-forming α1-subunit (Cav1, Cav2 and Cav3) or their sensitivity to be blocked by pharmaceutical agents (L, N, P/Q, R and T-type). Collectively, the high-voltage VGCCs include L-(Cav1.1, Cav1.2, Cav1.3, CaV1.4), P/Q-(Cav2.1), N-(Cav2.2) and R-(Cav2.3) type channels, while the low-voltage VGCCs include T-type (Cav3.1, Cav3.2, Cav3.3) channels. The high-voltage VGCCs typically form hetero multimers that consist of the channel-forming α1-subunit along with auxiliary β, α2δ, and γ-subunits.

**Figure 3:**
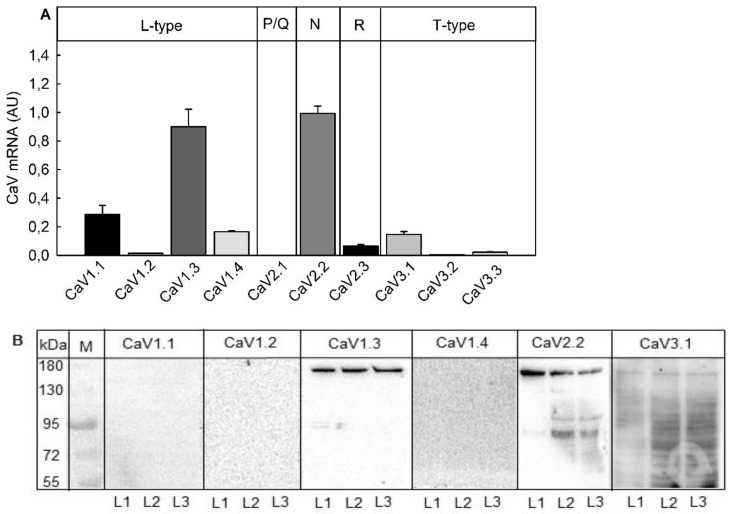
Expression of CaV in the reporter cell line. The expression of the CaVs was determined in differentiated SIMA cells stably expressing hPOMC1-26 GLuc. (**A**) Total RNA was isolated and relative mRNA expression of CaV was determined by RT-qPCR as described in the methods section. Values are means ± SEM of three independent mRNA preparations. (**B**) CaVprotein expression was determined in three independently produced cell lysates (L1, L2 and L3) with specific antibodies by Western blot.

Before the potential of VGCC inhibitors to block luciferase release was assessed in SIMA-hPOMC1-26-GLuc cells, the expression profile of VGCC channel-forming α1-subunits was analyzed in the reporter cell line both at the mRNA and protein level (Figure 3). qPCR analysis revealed that SIMA-hPOMC1-26-Gluc cells express VGCC in the descending order: CaV1.3 (L-type) = CaV2.2 (N-type) >> CaV1.1 (L-type) > CaV1.4 (L-type) = CaV3.1 (T-type) > CaV2.3 (R-type) > CaV3.3 (T-type) = CaV1.2 (L-type) > CaV2.1 (P/Q-type) (Figure 3A). On the protein level, only antibodies against the α1-subunits CaV1.3 (L-type) und CaV2.2 (N-type) detected proteins of the estimated molecular weight around 170–180 kDa by Western blot analysis in lysates of SIMA-hPOMC1-26-GLuc cells (Figure 3B). Therefore, it seems that SIMA-hPOMC1-26-GLuc cells mainly express VGCC from the L-type and N-type.

**Figure 4:**
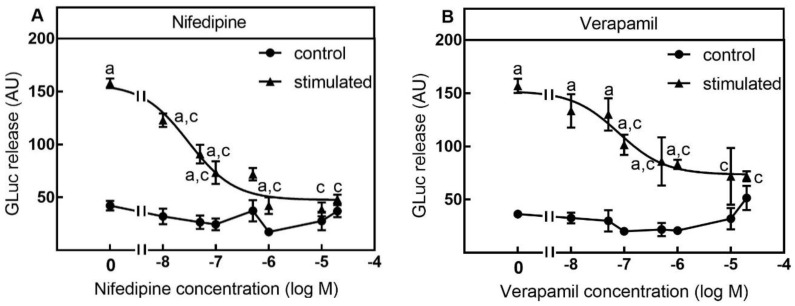
Inhibition of luciferase release by L-Type VGCC inhibitors. SIMA cells stably expressing hPOMC1-26 GLuc were cultured and differentiated as described in the methods section. After removing the medium, cells were washed with fresh medium and incubated in differentiation medium with different concentrations of L-type VGCC inhibitors nifedipine (**A**) or verapamil (**B**) for 10 min. Cells were then incubated for three minutes with non-depolarizing (Na^+^, control) or depolarizing (K_+_, stimulated) balanced salt solution in the presence of different nifedipine (**A**) or verapamil (**B**) concentrations. Cell culture supernatants were centrifuged, and luciferase activity was determined in the cell culture supernatants. Values are means ± SEM of at least three independent experiments. Statistics: Student’s t-test for unpaired samples, a: > control buffer without nifedipine or verapamil: c: < control or stimulation buffer without nifedipine or verapamil; *p* < 0.05.

In line with the VGCC expression profile K_+_-depolarization-stimulated luciferase release was significantly inhibited by the L-type VGCC inhibitors nifedipine and verapamil which were used as antihypertensive agents [15]. Nifedipine inhibited luciferase release with an EC50 of 33 nM (Figure 4A). The inhibition was significant from 10 nM and higher, and maximal at a concentration of 10 µM (Figure 4A). Similar to nifedipine, verapamil inhibited luciferase release with an EC50 of 79 nM which was significant from 100 nM and higher, and maximal at a concentration of 10 µM (Figure 4B).

At a concentration of 10 µM both nifedipine and verapamil decreased K^+^-stimulated luciferase release to the level of the unspecific release. Therefore, it appears that a major part of K^+^-depolarization stimulated GLuc release was L-type VGCC dependent and SIMA-hPOMC1-26-GLuc is a useful tool for the screening of neuronal L-type VGCC modulators.

**Figure 5:**
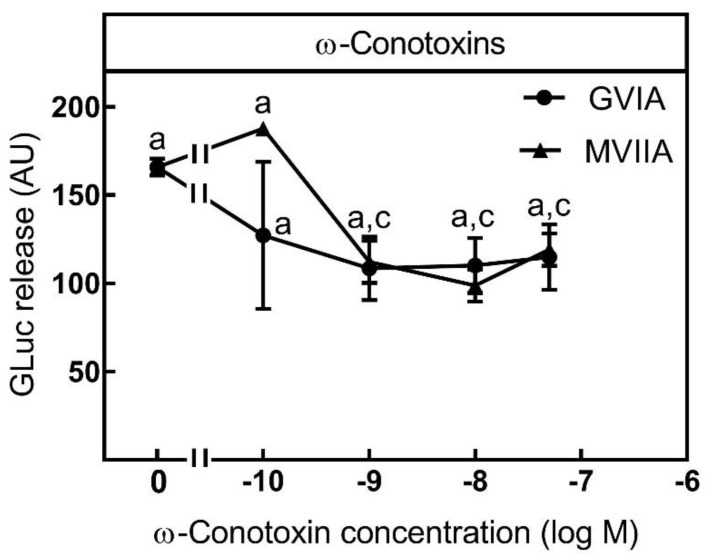
Inhibition of luciferase release by N-Type VGCC inhibitors. SIMA cells stably expressing hPOMC1-26 GLuc were cultured and differentiated as described in the methods section. After removing the medium, cells were washed with fresh medium and incubated in differentiation medium with different concentrations of N-type VGCC inhibitors ω-conotoxin GVIA or ω-conotoxin MVIIA for 10 min. Cells were then incubated for three minutes with non-depolarizing (Na^+^, control, not shown) or depolarizing (K^+^, stimulated) balanced salt solution in the presence of different ω-conotoxin concentrations. Cell culture supernatants were centrifuged, and luciferase activity was determined in the cell culture supernatants. Values are means ± SEM of at least three independent experiments. Statistics: Student’s t-test for unpaired samples, a: > control buffer without ω-conotoxins: c: < stimulation buffer without ω-conotoxins; *p* < 0.05.

The VGCC expression profile of SIMA-hPOMC1-26-GLuc also showed high expression of N-type VGCC, CaV2.2. In line with this, N-type VGCC inhibitors ω-conotoxins GVIA and MVIIA from marine cone snails decreased K^+^-depolarization stimulated luciferase release significantly and maximally at a concentration of 1 nM (Figure 5).

By contrast, at 1 nM neither ω-conotoxin affected unspecific luciferase release (not shown). In contrast to the L-type VGCC inhibitors nifedipine and verapamil, which showed a 55% reduction of luciferase release at a concentration of 10 µM, maximal ω-conotoxin-induced inhibition was only 30% of K^+^-stimulated luciferase release (Figure 5). Therefore, it appears that a part of K^+^-depolarization stimulated luciferase release was N-type VGCC dependent and SIMA-hPOMC1-26-GLuc cells are a suitable tool for the screening of neuronal N-type VGCC modulators.

**Figure 6:**
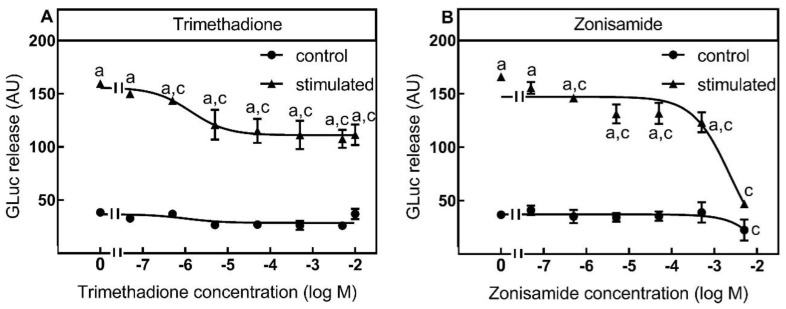
Inhibition of luciferase release by T-Type VGCC inhibitors. SIMA cells stably expressing hPOMC1-26 GLuc were cultured and differentiated as described in the methods section. After removing the medium, cells were washed with fresh medium and incubated in differentiation medium with different concentrations of T-type VGCC inhibitors trimethadione (**A**) or zonisamide (**B**) for 10 min. Cells were then incubated for three minutes with non-depolarizing (Na^+^, control) or depolarizing (K+, stimulated) balanced salt solution in the presence of different trimethadione (**A**) or zonisamide (**B**) concentrations. Cell culture supernatants were centrifuged, and luciferase activity was determined in the cell culture supernatants. Values are means ± SEM of at least three independent experiments. Statistics: Student’s t-test for unpaired samples, a: > control buffer without trimethadione or zonisamide: c: < control or stimulation buffer without trimethadione or zonisamide; *p* < 0.05.

In contrast to L-type and N-type VGCC the expression level of T-type VGCCs in SIMA-hPOMC1-26-GLuc cells was low (Figure 3). Surprisingly, K^+^-depolarization stimulated luciferase release was also significantly inhibited by the T-type VGCC inhibitors trimethadione and zonisamide, which are used in the therapy of neuronal diseases such as Parkinson and epilepsy [16]. Both trimethadione and zonisamide inhibited K^+^-depolarization mediated luciferase release significantly from 50 nM and higher, maximal at a concentration of 50 µM and left the unspecific release unaffected (Figure 6A,B).

Similar to the ω-conotoxins GVIA and MVIIA, trimethadione inhibited only 30% of the stimulated luciferase release (Figure 6A,B). In contrast to trimethadione, zonisamide was toxic for SIMA-hPOMC1-26-GLuc cells at a concentration of 5 mM and strongly suppressed unspecific and stimulated luciferase release (Figure 6B). Therefore, while expression level of T-type VGCC was low at the mRNA and protein level, it seemed that a small part of K^+^-depolarization stimulated luciferase release was T-type VGCC dependent and SIMA-hPOMC1-26-GLuc cells can also be used for the analysis of T-type mediators.

**Figure 7:**
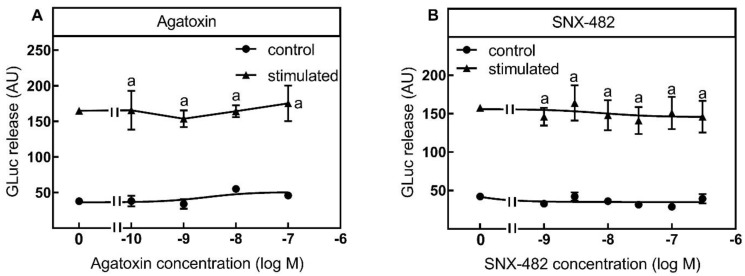
No inhibition of luciferase release by P/Q and R-Type VGCC inhibitors. SIMA cells stably expressing hPOMC1-26 GLuc were cultured and differentiated as described in the methods section. After removing the medium, cells were washed with fresh medium and incubated in differentiation medium with different concentrations of P/Q-type VGCC inhibitor agatoxin (**A**) or R-type VGCC inhibitor SNX-482 (**B**) for 10 min. Cells were then incubated for three minutes with non-depolarizing (Na^+^, control) or depolarizing (K^+^, stimulated) balanced salt solution in the presence of different agatoxin (**A**) or SNX-482 (**B**) concentrations. Cell culture supernatants were centrifuged, and luciferase activity was determined in the cell culture supernatants. Values are means ± SEM of at least three independent experiments. Statistics: Student’s t-test for unpaired samples, a: > control buffer without agatoxin or SNX482; *p* < 0.05.

According to their very low expression level, both the P/Q-type inhibitor agatoxin from the spider Agelonopsis aperta (Figure 7A) and the R-type VGCC inhibitor SNX-482 from the spider Hysterocrates gigas (Figure 7B) did not inhibit the luciferase release under control and stimulated conditions.

To exclude cytotoxicity, the impact of all VGCC inhibitors at the highest concentration employed in the release assays was tested in the AlamarBlue assay, based on the conversion of resazurin to the fluorogenic resorufin by viable cells. None of the toxins reduces cell viability (Supplementary Figure S1).

## 3. Discussion

### 3.1. Suitability of the Assay for Compounds Stimulating Neurotransmitter Release

Muscarinic acetylcholine receptor: In two former projects, release of the reporter enzyme GLuc was stimulated by a high K^+^-depolarization buffer and entry of extracellular Ca^2+^ into the reporter cell line [10,11]. One aim of this study was to analyze if GLuc can also be released by a Ca^2+^-increase from intracellular pools. Muscarinic acetylcholine receptors, a member of class I, seven transmembrane G-protein-coupled receptors (GPCRs), comprise five distinct subtypes, denoted as muscarinic M1, M2, M3, M4, and M5 receptors [17,18,19,20]. Whereas M2 and M4 receptors are Gi-coupled and inhibit cAMP-formation, M1, M3 and M5 receptors are coupled to Gq and increased intracellular calcium concentration from the sarcoplasmatic reticulum via phospholipase C activation and IP3-formation. Carbachol, a non-selective muscarinic acetylcholine receptor agonist, increases intracellular calcium concentration and neurotransmitter release [21]. In line with this, carbachol provoked the release of the reporter enzyme GLuc from SIMA-hPOMC1-26-GLuc cells in a dose-dependent manner and the release was completely blocked by the muscarinic acetylcholine receptor antagonist atropine. At high concentrations, carbachol-mediated GLuc release reached the same level as GLuc release stimulated by high K^+^-depolarization.

As dysfunction in the cholinergic system has been identified in various neuronal diseases, such as Parkinson and epilepsy [22,23], antagonists of the muscarinic system remain of great interest as potential lead CNS drug substances. The tropane alkaloids scopolamine and hyoscyamine are widely used as anticholinergic drugs [24]. Scopolamine has also been used in the treatment of motion sickness for a long time [25]. The drawbacks of scopolamine are the manifold central and peripheral nervous system side effects, as scopolamine is not receptor subtype specific. Development of new selective and potent muscarinic acetylcholine receptor antagonists either by de novo synthesis or screening of animal venoms is of note in the treatment of neuronal diseases [26]. Screening for anti-cholinergic drugs includes the structure guided development of M3 receptor specific antagonists [27] and the identification of M1 vs. M3 receptor selective drugs [28]. New antagonists were analyzed either by radioligand-binding studies with the membrane of M1-5 receptor transfected CHO cells or by functional studies (IP-formation and ß-arrestin recruitment) with receptor-overexpressing CHO and HEK293 cells. However, these models might not directly mirror the real functional read-out of anticholinergic drugs, the modulation of neurotransmitter release. To overcome this problem, our cell-based SIMA-hPOMC1-26-GLuc cell model may be a useful tool to verify basic screening of muscarinic acetylcholine receptor antagonist because a.) receptor expression level and expression profile is more similar to neuronal cells than in CHO or HEK293 cells and b.) the last functional step of signal transduction, the neurotransmitter release, is determined in the SIMA hPOMC1-26-GLuc cell model.

α-latrotoxin: The second aim of the study was to analyze whether calcium-channel forming toxins can stimulate GLuc release from SIMA-hPOMC1-26-GLuc cells instead of high-K^+^- depolarization buffer. α-Latrotoxin (α-LTX), a neurotoxin from black widow spider venom triggers neurotransmitter release by synaptic vesicle exocytosis from presynaptic nerve terminals. It is the main toxic component in the venom of black widow spiders, whose bite leads to latrodectism, a syndrome consisting of muscle pain, abdominal cramps and raised blood pressure [29]. α-LTX has been an extremely useful tool in the analysis of synaptic signal transmission as α-LTX acts very selectively on presynaptic nerve terminals [12]. The action of α-LTX is mediated by two distinct mechanisms: First, α-LTX can bind to the receptor molecule latrophilin, leading to insertion of α-LTX in the plasma membrane. This stimulates exocytosis of classical neurotransmitters such as glutamate and acetylcholine in a calcium-independent manner 12]. Second, α−LTX interacts with the protein neurexin to form a permanently open Ca^2+^-channel, which leads to the release of catecholamines in a calcium-dependent manner [12].

In the present study, in low nM concentrations α-LTX stimulated release of the reporter GLuc under control conditions, but did not affect GLuc release by high K^+^-depolarization (Figure 2). At the highest concentration used (5 nM), α-LTX mediated GLuc release did not differ from high K^+^-depolarization induced release. As the α-LTX stimulated GLuc release was completely blocked by EGTA, the mechanism of α-LTX dependent GLuc release might reflect neurexin and calcium-dependent release of catecholamines rather than latrophilin and Ca^2+^-independent release of classical neurotransmitters.

Interestingly, α-LTX not only stimulates a massive exocytosis of neurotransmitters but also causes an acute and complete degeneration of motor axon terminals, followed by a rapid recovery [30]. By contrast, botulinum toxins induce a long-lasting paralysis without nerve-terminal degeneration. In a former study, it was shown that injection of α-LTX in mouse muscles which were paralyzed with BoNT/A accelerates the recovery of neurotransmission from several months to a few days [31]. This interplay of both toxins can bring more insights into the mechanisms of peripheral human pathologies due to degeneration of motor axon terminals. As the SIMA-hPOMC1-26-Gluc cell line can easily measure the action of BoNT and α-LTX, it might be a useful tool to analyze the interaction of both toxins in their regulation of neurotransmitter release.

### 3.2. Suitability of the Assay for Compounds Inhibiting Neurotransmitter Release

The last aim of the study was to analyze if the GLuc release from SIMA-hPOMC1-26-GLuc cells by high K^+^-depolarization can be inhibited by VGCC inhibitors rather than by BoNTs, which has previously been demonstrated [11]. Voltage dependent Ca^2+^-channels are a group of voltage-gated ion channels with a permeability for Ca^2+^-ions. They are formed as a complex of different subunits: α1, α2δ, β1-4, and γ, where the α1subunit forms the ion conducting pore. According to their calcium pore forming α1-subunit (CaV) VGCCs can be classified in several types, the L-type (CaV1.1–CaV1.4), P/Q-type (CaV2.1), N-type (CaV2.2), R-type (CaV2.3) and T-type (CaV3.1–CaV3.3). They can be discriminated by their inhibition by different neurotoxins and neuro-pharmaceutical inhibitors. N-type VGCC are interesting therapeutic targets for the treatment of nociceptive pain whereas T-type VGCCs channel blockers are used as antiepileptic drugs [16,32]. L-Type VGCC inhibitors such as dihydropyridines have been used as antihypertensive agents for a long time, but block both CaV1.2 and CaV1.3 VGCCs [33]. The newer Cav1.3 VGCCs specific dihydropyridine derivate isradipine was shown to be neuroprotective in a mouse model of Parkinson disease [34] and was discussed as a potential strategy for the treatment of Alzheimer disease [35].

As VGCCs are interesting targets for the treatment of neuronal diseases, SIMA-hPOMC1-26-Gluc cells were first analyzed for VGCC expression. The highest expression on the mRNA and protein level was measured for L-type CaV1.3 and N-type CaV2.2, whereas T-type CaV3.1 and L-type CaV1.1 were detected only on the mRNA level. The CaV expression profile was nearly identical to the profile in the neuroblastoma cell line SH-SY5Y [36]. In line with the expression profile of L-Type inhibitors verapamil and nifedipine, N-type inhibitors ω-conotoxins GVIA and MVIIA, and T-type inhibitors zonisamide and trimethadione inhibited GLuc release induced by high-K^+^-depolarization. In contrast to the high expression of N-type CaV2.2, inhibitors of L-type CaV showed the strongest inhibition of GLuc release, whereas ω-conotoxins, as well as zonisamide and trimethadione, were less active. Since SIMA-hPOMC1-26-GLuc cells do not express L-type CaV1.1, CaV1.2 and CaV1.4, the cell-based assay may be suitable for functional screening for CaV1.3 inhibitors.

### 3.3. Cytotoxicity of Compounds Inhibiting Neurotransmitter Release

Cytotoxicity could affect the assay in two ways: Cell lysis could increase the non-specific release. This would yield false positive results in assays, which test, for example, calcium channel activators. On the other hand, cytotoxicity could non-specifically interfere with the fusion of neuro-secretory vesicles. This would yield false positive results, for example, for calcium channel blockers. However, at the maximal concentration used in the release assays, none of the VGCC inhibitors tested showed cytotoxic side effects.

## 4. Conclusions

In conclusion, the newly established cell-based assay may represent a versatile tool for the analysis of neurotoxins and neuroactive pharmaceuticals which act by the modulation of intracellular calcium-concentration. The applications range from compounds which stimulate neurotransmitter release to inhibiting compounds.

## 5. Materials and Methods

### 5.1. Materials

All chemicals were purchased from commercial sources indicated throughout the text. Oligonucleotides were custom-synthesized by Eurofins Operon (Ebersberg, Germany) or Biolegio (Nijmegen, The Netherlands). Neurotoxins and neuroactive pharmaceuticals used: carbachol, atropine, verapamil, nifedipine, thrimethadione and zonisamide were from Sigma-Aldrich (Taufkirchen, Germany); α-Latrotoxin (ALX-630-027) was from Enzo Life Science (Lörrach, Germany); ω-conotoxins GVIA and MVIIA were from Alamone labs (Jerusalem, Israel); agatoxin and SNX-482 were from tebu-bio (Offenbach, Germany). Antibodies used were: CaV1.1 (sc-514685), CaV1.2 (sc-398433), CaV1.3 (sc-515679), CaV1.4 (sc-517005) and CaV2.2 (sc-271010) were from SantaCruz Biotechnology (Heidelberg, Germany) and CaV3.1 (Acc-021) was from Alamone Labs.

### 5.2. Cell Culture

Generation of the stably transfected neuroblastoma cell line SIMA hPOMC1-26 GLuc has been described previously [10]. Non-transfected SIMA cells were originally from DSMZ, (Braunschweig, Germany). Cells were cultured in RPMI 16040 medium supplemented with 10% (*v*/*v*) heat-inactivated fetal calf serum (FCS), 2 mM stable L-alanyl-L-glutamine and penicillin (100 U/mL)/streptomycine (100 µg/mL) as antibiotics.

### 5.3. Luciferase Release from Cells Treated with Neurotoxins or NeuroActive Pharmaceuticals

For release experiments SIMA-hPOMC1-26-GLuc cells were differentiated in poly l-lysine coated 96-well plates (5 × 103–5 × 104 cells/well) with differentiation medium (RPMI 1640 supplemented with 1 x B27 supplement, 1 x N2 supplement, 2 mM L-alanyl-L-glutamine, 1 mM non-essential amino-acids, 10 mM 4-(2-hydroxyethyl)-1-piperazineethanesulfonic acid (HEPES) and penicillin (100 U/mL)/streptomycin (100 µg/mL)) for 96 h with a medium change after 48 h. Subsequently, cells were preincubated with 100 µL fresh medium in the absence or presence of VGCC inhibitors for 10 min at 37 °C. The medium was aspirated and GLuc release was stimulated with 100 µL/well control (20 mM Hepes pH 7.4, 136 mM NaCl, 4.7 mM KCl, 1.25 mM CaCl_2_ and 1.25 mM MgSO_4_) or depolarization buffer (20 mM Hepes pH 7.4, 40.7 mM NaCl, 100 mM KCl, 1.25 mM CaCl_2_ and 1.25 mM MgSO_4_) in the absence or presence of carbachol or α-latrotoxin for 3 min or 5 min (α-latrotoxin) at 37 °C. The supernatant was transferred into reaction vials and centrifuged at 100× *g* for 3 min to remove detached cells. To determine GLuc activity 20 µL of the supernatant was mixed with 100 µL luciferase substrate solution and the luminescence was measured using Fluostar Optima. GLuc release was normalized to GLuc activity in remaining lysed cells and the mean of GLuc activity in untreated control and stimulated cells was set to 100% (AU).

### 5.4. Real-Time RT-PCR

Total RNA from differentiated SIMA hPOMC1-26-GLuc cells was isolated using peqGold Total RNA Kit (Peqlab, Germany). 1–2 µg total RNA was reverse transcribed into cDNA using an oligo dT as a primer and an M-MuLV Reverse Transcriptase (Thermo Scientific, Darmstadt, Germany). Hot start real-time PCR for the quantification of each transcript was carried using 2 x Maxima SybrGreen qPCR mix (Thermo Scientific), 0.25 µM of each primer and 2.5–5 µL of cDNA, which was diluted 1:10. PCR was performed with an initial enzyme activation step at 95 °C for 10 min, followed by 42 cycles of denaturation at 95 °C for 30 sec, annealing at 57 °C for 30 sec and extension at 72 °C for 1 min in a real-time DNA thermal cycler (CFX96™, 10 µL reaction volume, BIO-RAD; Munich, Germany). The oligonucleotides used are listed in Table 1. The expression levels of VGCC were calculated relative to GAPDH as a reference gene.

### 5.5. Western Blot Analysis

SIMA hPOMC1-26-GLuc cells were lysed in Lämmli sample buffer (80 mM Tris/HCl pH 6.8, 2% (*w*/*v*) SDS, 5% (*w*/*v*) glycerol, 0.025% *w*/*v* bromophenol blue and 5% (*v*/*v* 2-mercatoethanol) homogenized by sonication. Insoluble material was removed by centrifugation (10,000× *g*, 15 min, 4 °C). Proteins were resolved by SDS-PAGE and transferred to a polyvinylidene difluoride (PVDF) membrane. Membranes were blocked in 5% non-fat dry milk in 20 mM Tris, 136 mM NaCl and 0.1% (*v*/*v*) TWEEN 20 (Polyoxyethylenesorbitan monolaurate, TBS/Tween) for 1 h at room temperature and incubated with anti CaV antibodies in TBS/Tween containing 5% bovine serum albumin overnight at 4 °C and a horseradish-peroxidase-conjugated anti-rabbit or anti-mouse IgG for 2 h at room temperature. Visualization of immune complexes was performed using chemoluminescence reagent Clarity Western ECL (BIO-RAD, Feldkirchen, Germany).

### 5.6. Cytotoxicity Assay

For the determination of overall cytotoxicity differentiated SIMA-hPOMC1-26-GLuc cells were preincubated with 100 µL fresh medium in the absence or presence of VGCC inhibitors for 10 min at 37 °C. The medium was aspirated and 100 µL/well resazurin medium (90% (*v*/*v*) differentiation medium + 10% (*v*/*v*) 1 mg/mL resazurin in PBS) was added. Fluorescence of resorufin, liberated from the chromogen by vital cells only was determined in the Fluostar Optima microreader (BMG-Labtech, Ortenberg, Germany) with 530 nm excitation and 590 nm emission wavelength filters. The increase of fluorescence was monitored every 30 min for 2 h, the slope of the fluorescence increase was determined in the linear part.

## Figures and Tables

**Table 1:** Oligonucleotide primers used for realtime qPCR.

Gene	Forward	Reverse
GAPDH CaV 1.1 CaV 1.2 CaV 1.3 CaV 1.4 CaV 2.1 CaV 2.2 CaV 2.3 CaV 3.1 CaV 3.2 CaV 3.3	5′-TGATGACATCAAGAAGGTGG 5′-ACCATTGAGGAAGAGGCAGC 5′-TGCCCTTGCATCTGGTTCAT 5′-CCCAGGCAGAAACATCGACT 5′-CTTGGTGGAGGCTGTGCTTA 5′-CCTGAGCATGACCACCCAAT 5′-TACAAGACGGCCAACTCCTC 5′-AGACGCTCACTTTCGAAGCA 5′-GCTGGATGAGCAGAGGAGAC 5′-CTCAGGGCTTCCTGGACAAG 5′-GAAGAGATGAGGGTCGCAGG	5′-TTACTCCTTGGAGGCCATGT 5′-CATAGGCGACATTGGCGTTG 5′-ATCAAGACCGCTTCCACCAG 5′-CTGCCATGATCTGTTGCTGC 5′-TATTGAGCAGTTGGGGAGGG 5′-CATGTGCTCTCGGCCCTC 5′-TCAGGGAGGACACGTAGGAA 5′-TTGTTGACAGCCCCACACAT 5′-ATCTTTCTTTGGGGAGGGCG 5′-CCGTCCAAGAAAGGGTCTCC 5′-GCCAGAATCCCAGAGCATCA

Accession numbers for the genes were: GAPDH (AB062273), CaV 1.1 (NM_000069.2), CaV 1.2 (NM_199460.3), CaV 1.3 (NM_000720.3), CaV 1.4 (NM_005183.3), CaV 2.1 (NM_000068.3), CaV 2.2 (NM_000718.3), CaV 2.3 (NM_001205293.1), CaV 3.1 (BC110995.1), CaV 3.2 (NM_021098.2), CaV 3.3 (NM_021096.3).

## Data Availability

Original data and Excel files are available on request.

## Supplementary Materials

The following are available online at https://www.mdpi.com/article/10.3390/toxins13040247/s1, Figure S1 Cytotoxicity assay with SIMA-hPOMC1-26-GLuc cells and VGCC inhibitors. Differentiated SIMA-hPOMC1-26-GLuc cells were preincubated with 100 µL fresh medium in the absence or presence of VGCC inhibitors or 0.1% (*v*/*v*) Triton X-100 for 10 min at 37 °C. The medium was aspirated and 100 µL/well resazurin containing medium was added. Fluorescence of resorufin, generated by resazurin reduction by vital cells only was determined in the Fluostar Optima microreader with 530 nm excitation and 590 nm emission wavelength filters. The increase of fluorescence was monitored every 30 min for 2 h, the slope of the fluorescence increase was determined in the linear part. Data are means ± SEM of 3-8 independent determinations performed in triplicate. Statistics: Student’s t-test for unpaired samples. a: < naive, *p* < 0.05.
