# Supplementary material for: Cell-Based Reporter Release Assay to Determine the Activity of Calcium-Dependent Neurotoxins and Neuroactive Pharmaceuticals
Source: Toxins (Basel). 2021 Mar 30;13(4):247. doi: 10.3390/toxins13040247 (PMC8066854; doi:10.3390/toxins13040247)
Supplement: Supplementary file 1 [file toxins-13-00247-s001.pdf]

Supplementary Materials: Cell-Based Reporter Release Assay to Determine the Activity of Calcium-Dependent Neurotoxins and Neuroactive Pharmaceu-ticals

Andrea Pathe-Neuschäfer-Rube, Frank Neuschäfer-Rube and Gerhard P. Püschel

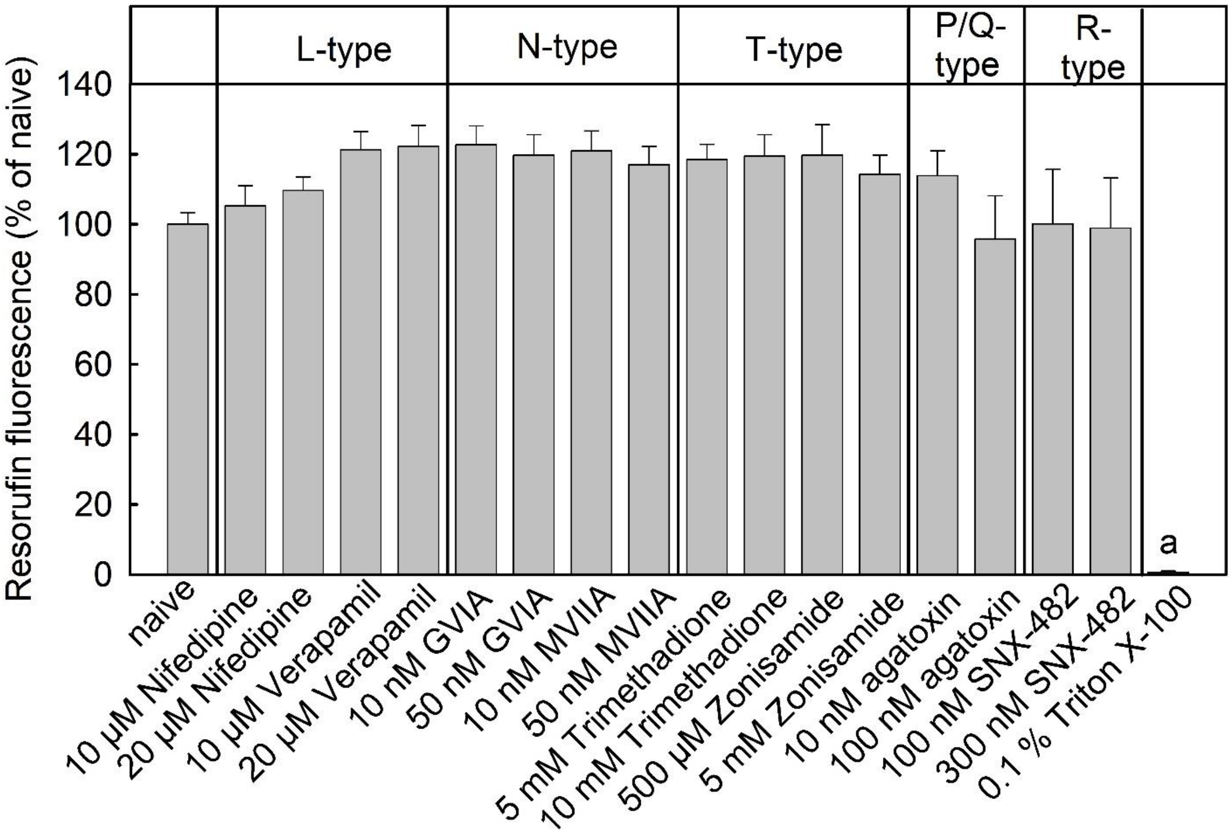

Figure S1. Cytotoxicity assay with SIMA-hPOMC1-26-GLuc cells and VGCC inhibitors. Differenti-ated SIMA-hPOMC1-26-GLuc cells were preincubated with 100 µL fresh medium in the absence or presence of VGCC inhibitors or 0.1% (*v*/*v*) Triton X-100 for 10 min at 37 °C. The medium was aspirated and 100 µL/well resazurin containing medium was added. Fluorescence of resorufin, generated by resazurin reduction by vital cells only was determined in the Fluostar Optima microreader with 530 nm excitation and 590 nm emission wavelength filters. The increase of fluorescence was monitored every 30 min for 2 h, the slope of the fluorescence increase was determined in the linear part. Data are means ± SEM of 3-8 independent determinations performed in triplicate. Statistics: Student's t-test for unpaired samples. a: < naive, $p < 0,05$.
